# Prognostic Value of Tumor Volume, Tumor Volume Reduction Rate and Magnetic Resonance Tumor Regression Grade in Rectal Cancer

**DOI:** 10.3390/medicina59122194

**Published:** 2023-12-18

**Authors:** Ingrida Pikūnienė, Vestina Strakšytė, Algidas Basevičius, Justas Žilinskas, Rita Ambrazienė, Rasa Jančiauskienė, Žilvinas Saladžinskas

**Affiliations:** 1 Department of Radiology, Hospital of Lithuanian University of Health Sciences Kauno Klinikos, LT-50161 Kaunas, Lithuaniaalgidas.basevicius@kaunoklinikos.lt (A.B.); 2 Department of Surgery, Hospital of Lithuanian University of Health Sciences Kauno Klinikos, LT-50161 Kaunas, Lithuania; justas.zilinskas@lsmu.lt (J.Ž.); zilvinas.saladzinskas@kaunoklinikos.lt (Ž.S.); 3 The Institute of Oncology of the Faculty of Medicine, Lituanian University of Health Sciences, LT-50161 Kaunas, Lithuania; rita.ambraziene@gmail.com (R.A.);

**Keywords:** rectal cancer, tumor volume, tumor volume reduction rate, magnetic resonance tumor regression grade, pathological tumor regression grade

## Abstract

*Background and Objectives*: Rectal cancer poses significant treatment challenges, especially in advanced stages. Radiologic assessment, particularly with MRI, is critical for surgeons and oncologists to understand tumor dynamics and tailor treatment strategies to improve patient outcomes. The purpose of this study was to correlate MRI-based tumor volumetric and tumor regression grade analysis in patients with advanced rectal cancer, assessing the impact of preoperative chemotherapy (CT) alone or chemoradiotherapy (CRT) on surgical technique choices. *Materials and Methods*: Between 2015 and 2022, a prospective study was enrolled, including a cohort of 89 patients diagnosed with rectal cancer at stage II or III. The participants were divided into two distinct therapy groups, ensuring an equal distribution with a ratio of 1:1. The initial group was treated with the contemporary preoperative chemotherapy protocol FOLFOX4. In contrast, the alternative group received conventional preoperative chemoradiotherapy. Before surgery, each patient underwent a rectal MRI scan at 1.5 T, including T2-weighted and diffusion-weighted imaging (DWI) sequences. *Results*: The CT group showed a 36.52% tumor volume reduction rate (TVRR), and the CRT group showed 54.87%, with varying magnetic resonance and pathological tumor regression grades (mrTRG and pTRG). Analysis revealed a significant interaction between mrTRG and tumor volumetrics (volume and VRR) in both groups, especially CRT, underscoring the complexity of tumor response. Both treatment groups had similar initial tumor volumes, with CRT displaying a higher TVRR, particularly in higher pathological TRG (3/4) cases. This interaction and the strong correlation between mrTRG and pTRG suggest mrTRG’s role as a non-invasive predictor for treatment response, highlighting the need for personalized treatment plans. *Conclusions*: Rectal tumor volume, volume reduction rate, and mrTRG are not just abstract measures; they are concrete indicators that have a direct and practical impact on surgical decision-making, planning, and prognosis, ultimately influencing the quality of care and life expectancy of patients with rectal cancer.

## 1. Introduction

Colorectal cancer is the third most frequently diagnosed cancer, accounting for approximately 10% of all cancer incidences and standing as the world’s second primary cause of death due to cancer [1].

Rectal cancer presents a major health challenge because of its high incidence and mortality rate, posing complex issues for its effective management. Despite progress in treatment modalities, patient prognosis remains highly variable and is influenced by a spectrum of factors, including the initial size of the tumor and the rate at which it decreases during treatment. Magnetic resonance imaging (MRI) provides crucial information concerning clinical prognosis and preoperative assessment [2]. Tumor volume and tumor volume reduction rates have been identified as pivotal prognostic tools that promise to enhance treatment planning and patient care strategies [3]. Next, we can add one more prognostic factor: magnetic resonance tumor regression grade (mrTRG). Although the limitations and agreement of mrTRG with pTRG are still under investigation, it is already gaining attention for its potential to closely monitor and guide therapeutic interventions [4].

The accurate evaluation of the initial tumor volume is a crucial aspect of refining rectal cancer surgical techniques. It gives surgeons essential information about tumor size and spread, helping them select the optimal surgical approach, whether laparoscopic, robotic, minimally invasive, or open surgery [5]. In addition, this assessment is critical for identifying candidates suitable for conservative, organ-preserving approaches and the “watch-and-wait” strategy [6]. The location of the tumor and its proximity to vital structures significantly influence surgical decisions. Understanding tumor volume is also critical for predicting potential post-operative issues, including prolonged recovery time and risk of complications such as anastomotic leakage, bleeding, or infection [7]. This information is essential to effectively prepare patients and manage their expectations regarding recovery and potential changes in bowel function after surgery [8]. As a result, accurate knowledge of the initial tumor volume is a cornerstone in improving the safety, efficacy, and overall advancement of surgical methods to treat rectal cancer.

Furthermore, the tumor volume reduction rate, especially after neoadjuvant therapy, indicates the biological behavior of the tumor. A significant decrease in tumor size post-therapy is often a positive indicator, suggesting that the cancer is responsive to the treatment [9]. This responsiveness can lead to less extensive surgical interventions.

mrTRG offers critical insights into the probability of achieving a complete cancer resection with clear margins, a key factor in determining local recurrence and overall patient survival. Despite its value, the concordance between mrTRG and pTRG (pathological TRG) remains limited. Consequently, mrTRG cannot substitute for pTRG. There is a growing need for further research to enhance mrTRG’s effectiveness in identifying patients eligible for non-surgical treatment options and complementing pTRG for more nuanced post-operative risk stratification [10]. Additionally, the volume reduction rate might influence the surgery timing. Patients who show rapid response to treatment may undergo surgery sooner, whereas those with slower response rates may benefit from extended cycles of chemotherapy or radiation before undergoing surgery.

From a predictive standpoint, both the tumor volume reduction rate and mrTRG can guide the surgeon in discussing long-term outcomes with the patient. Data suggest that patients whose tumors significantly reduce in volume may have a better prognosis, which can be vital to post-operative care and surveillance strategies [11].

This nuanced understanding of tumor biology requires sophisticated imaging and analytical techniques, with modalities such as MRI playing pivotal roles in volumetric analysis [12]. Moreover, integrating these measurements into predictive models and treatment algorithms can customize therapeutic strategies, optimizing the balance between efficacy and toxicity for each patient.

The adoption of tumor VRR and mrTRG as standard prognostic tools in rectal cancer care faces several challenges. These include variability in measurement techniques, timing of assessments, and differences in interpretation among observers, which can affect the accuracy and consistency of these metrics. The underlying biological reasons why some tumors respond better to treatment while others resist it are not fully understood. Considering these challenges, our study aimed to explore the relationship between tumor volumetrics and tumor regression grade, as assessed by MRI, in patients with locally advanced rectal cancer.

## 2. Materials and Methods

### 2.1. Patients

This prospective study at the Kaunas Clinics of Lithuanian Health Science University from 2015 to 2022 involved 89 patients with stage II–III rectal cancer. The study was approved by the Regional Biomedical Research Ethics Committee (Protocol No.: BE-2-32) under ClinicalTrials.gov Identifier: NCT05378919. Each patient consented to participate.

This cohort, aged 37 to 83 years, was split equally into two groups: one received a preoperative FOLFOX4 chemotherapy regimen and the other received standard preoperative chemoradiotherapy. Inclusion criteria were adults with biopsy-confirmed adenocarcinoma, measurable tumors, ECOG 0–2, and no distant metastases. Exclusion was due to obstruction, previous lower abdomen radiation, another recent tumor, pregnancy, nursing, or significant comorbidities.

A total of 40 patients received FOLFOX4 over eight cycles, and 49 underwent chemoradiation. The response to treatment was monitored using MRI and cancer markers, influencing subsequent surgical decisions. Radiotherapy was added if the tumor persisted at the T4 or N(+) stage. Post-surgery, the chemotherapy group completed four more cycles if an R0 resection was achieved, whereas the control group received the Mayo regimen postoperatively. All the patients are presented in Table 1.

### 2.2. MRI Protocol

MRI scans were performed using a Siemens 1.5-T Magnetom Avanto scanner with surface coils. In the supine position, patients received pelvic MRI for initial cancer staging and again 6–8 weeks after treatment for response evaluation.

Patients fasted for 4–6 h and had a microenema before MRI. Butylscopolamine (20 mg IV) was administered to reduce bowel movement. Contrast-enhanced MRIs were performed using gadolinium-based agents (0.2 mL/kg) followed by a saline flush.

Imaging protocols involved T2-weighted sequences in multiple planes and axial diffusion-weighted imaging (DWI) with fat suppression. The DWI parameters included multiple b values and fat suppression to create detailed images and apparent diffusion coefficient (ADC) maps for tissue characterization.

### 2.3. MR Volumetry and Tumor Regression Grade (mrTRG and pTRG)

**Figure 1:**
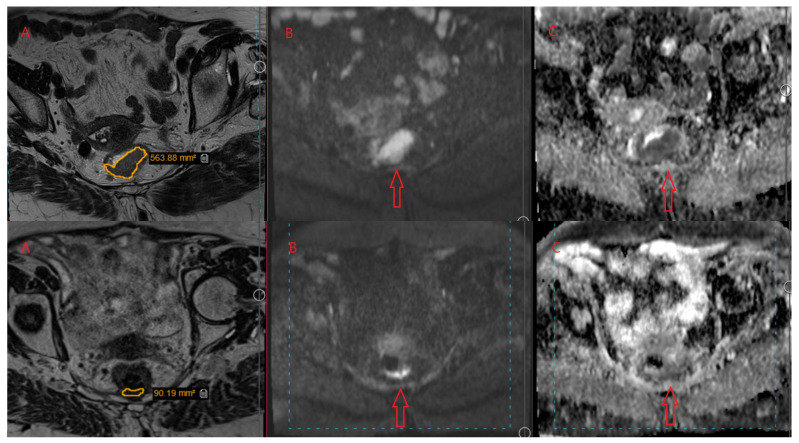
Represent MRI of rectal cancer before (**upper** pictures) and after treatment (**lower** pictures) in the same transverse section: Region of Interest (ROI) was sketched in yellow line on T2W images, marked tumor with red arrow in DWI images and ADC map images. (**A**) = T2W image; (**B**) = DWI image; (**C**) = ADC map image.

A radiologist with seven years of experience interpreting rectal MRI scans conducted the assessment without knowing the pathological results. Before and after treatment, high-resolution MRI was used to determine the tumor’s cross-sectional dimensions by assessing the T2-weighted sagittal and axial images. Figure 1 illustrates the corresponding ADC maps before and after CRT.

The tumor’s area in square centimeters (cm^2^) was measured in T2-weighted axial images. This was achieved by manually tracing the tumor boundaries on each image slice to define an irregular region of interest (ROI) (Figure 1). This area was multiplied by the slice interval (0.3 cm) to calculate the tumor volume. The process was repeated for every tumor slice, and the volumes were added together. To determine the extent of tumor shrinkage post-treatment, the percentage decrease in volume was calculated as follows:

Regression% = (volume2 − volume1) × 100/volume1.

The response to neoadjuvant CRT or FOLFOX4 in rectal cancer was evaluated using the mrTRG classification, which originates from the methodology of the MERCURY study group [13]. This scoring system is based on assessing residual tumor and fibrosis as seen on T2-weighted MRI. In addition, actual tumor specimens were examined by certified pathologists using the Dworak regression scale to gauge the effectiveness of the neoadjuvant chemotherapy (Table 2). Patients were categorized into two groups according to their different tumor responses to magnetic resonance findings. mrTRG1-2 was defined as a good responder, and mrTRG 3-4-5 was defined as a bad responder. The same categorization was applied to the different tumor responses of the surgical pathology. Stage pTRG3–4 was described as a good responder, and pTRG 0–1–2 was defined as a bad responder.

### 2.4. Statistical Analysis

Continuous data are presented as the mean with standard deviation or as a median range (Minimum–Maximum). The significance of the relationship between tumor volume reduction rate (TVRR) and patient characteristics was determined using the *t*-test or chi-square test. Logistic regression analysis was used to explore the association between TVRR and indicators of pathological response. Receiver operating characteristic (ROC) curves were generated, providing the area under the curve (AUC) and enabling the determination of sensitivity, specificity, and positive and negative predictive values at optimal threshold levels (balancing sensitivity and specificity). All analyses were conducted using SPSS (version 23.0). A *p*-value was established at <0.05 for significant results.

## 3. Results

### 3.1. Patients Characteristics

Our research involved 89 patients aged 37 to 83 years, with an average of 64.26 years (SD 9.165). Most patients were male (60, 67.4%), with females constituting 29 (32.6%) of the sample. We staged the tumor through MRI scans (T2W and DWI modalities) alongside pathological evaluations (Table 3). Among those receiving CT, males comprised 67.5% (27) and females 32.5% (13). Similarly, in the CRT control group, males represented 67.3% (33), and females represented 32.6% (16).

### 3.2. MR Volumetry, mrTRG and Pathological Results

The pathological results of all 89 study patients who underwent surgery are presented in Table 4. In the CT group, the pre-therapy volume was 48.24 cm^3^ (SD 17.67 cm^3^), and the post-treatment volume was 30.66 cm^3^ (SD 13.78 cm^3^), with a tumor volume reduction rate of 36.52% (SD 17.01%). In the CRT group, the volumes were 46.97 cm3 (SD 16.45 cm^3^), 20.54 cm^3^ (SD 10.41 cm^3^), and 54.87% (SD 19.57%), respectively.

**Figure 2:**
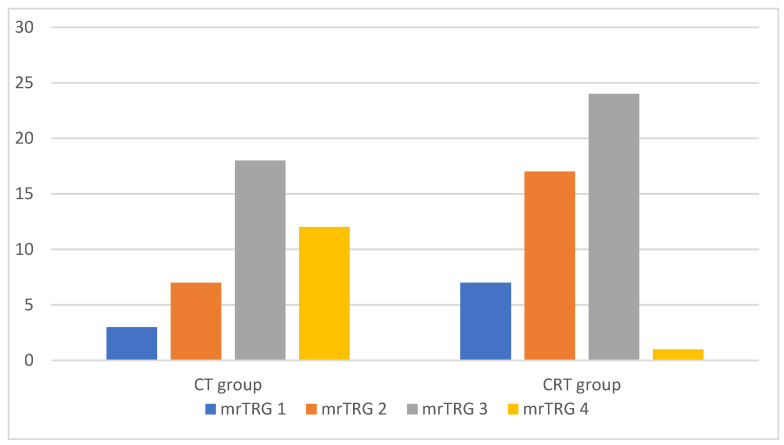
Diagram of both treatment groups according to magnetic resonance tumor regression grade. CT group = Chemotherapy group; CRT group = Chemoradiotherapy group; mrTRG1/2/3/4 = magnetic resonance tumor regression grade 1/2/3/4.

Within the CT group, mrTRG assessment showed 3 (7.5%) cases in mrTRG1, 7 (17.5%) in mrTRG2, 18 (45%) in mrTRG3, and 12 (30%) in mrTRG4. In the CRT group, the numbers were as follows: 7 (14.2%) cases in mrTRG1, 17 (34.7%) in mrTRG2, 24 (48.8%) in mrTRG3, and 1 (2.3%) in mrTRG4 (Figure 2).

**Figure 3:**
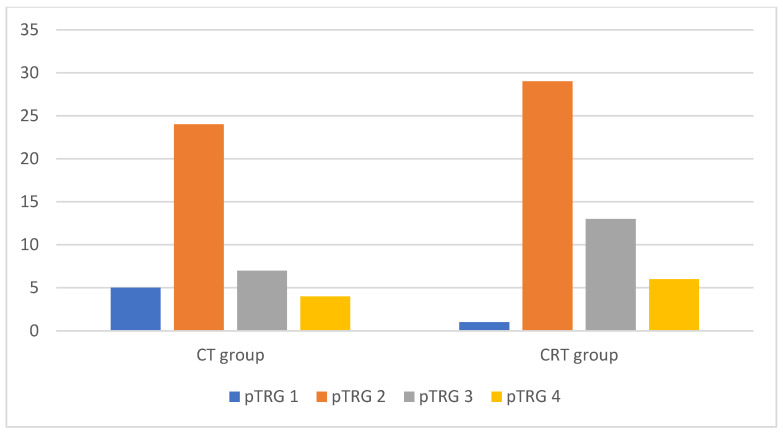
Diagram of both treatment groups according to pathological tumor regression grade. CT group = Chemotherapy group; CRT group = Chemoradiotherapy group; pTRG1/2/3/4 = pathological tumor regression grade 1/2/3/4.

CT group pathological TRG assessment showed 5 (12.5%) cases in TRG1, 24 (60%) cases in TRG2, 7 (17.5%) in TRG3, and 4 (10%) in TRG4. CRT group pathological TRG assessment showed 1 (2.3%) case in TRG1, 29 (59.2%) in TRG2, 13 (26.5%) in TRG3, and 6 (12%) in TRG4 (Figure 3).

### 3.3. Interaction of MR Volumetry, TVRR, mrTRG, and pTRG

In the study that assessed the effects of certain factors on the dependent variable, CT and CRT groups were examined using a two-factor analysis of variance with repeated measures (Table 5). This study aimed to determine the impact of tumor volume in cm^3^ and tumor volume reduction rate (VRR %), as well as mrTRG, on the outcome. It was found that neither mrTRG nor tumor measurements (volume or VRR) alone demonstrated a significant difference regarding the dependent variable, suggesting that these parameters did not independently influence the outcome within these two groups.

However, an interesting interaction between mrTRG and tumor volumetric measurements (volume and VRR) was observed, suggesting that the combined effect of mrTRG with tumor volume and VRR significantly impacted the dependent variable in both groups. This indicates that the relationship between tumor response and regression grade is complex and may influence how tumor characteristics interact. Furthermore, the interaction between mrTRG and tumor volumetrics in the CRT group was even more pronounced, with a highly significant impact on the dependent variable. This underscores the importance of considering the physical response of the tumor and regression grading when evaluating outcomes in this group.

Similar data were found according to pTRG (Table 6). In evaluating tumor response to treatment, CT and control CRT presented similar pre-therapy tumor volumes with no statistically significant difference (*p* = 0.351 for CT and *p* = 0.272 for CRT). This suggests that both groups started with comparable tumor burdens.

Post-therapy volumetry revealed a reduction in tumor size for both groups, but the differences were not statistically significant within the groups (*p* = 0.187 for CT and *p* = 0.117 for CRT). It indicates that while both therapies effectively reduced tumor volume, there was considerable variability within each treatment group that could not be attributed to the type of treatment alone.

However, TVRR showed a significant difference in both groups (*p* < 0.001 for CT and CRT). The CT group had an average TVRR of 46.18% (SD 25.36%). In comparison, the CRT group had a notably higher TVRR of 65.28% (SD 21.18%) for the 3/4 pTRG category and a lower TVRR of 48.28% (SD 15.48%) for the 0/1/2 pTRG category, indicating a more pronounced response in the CRT group, particularly in the 3/4 pTRG category.

**Figure 4:**
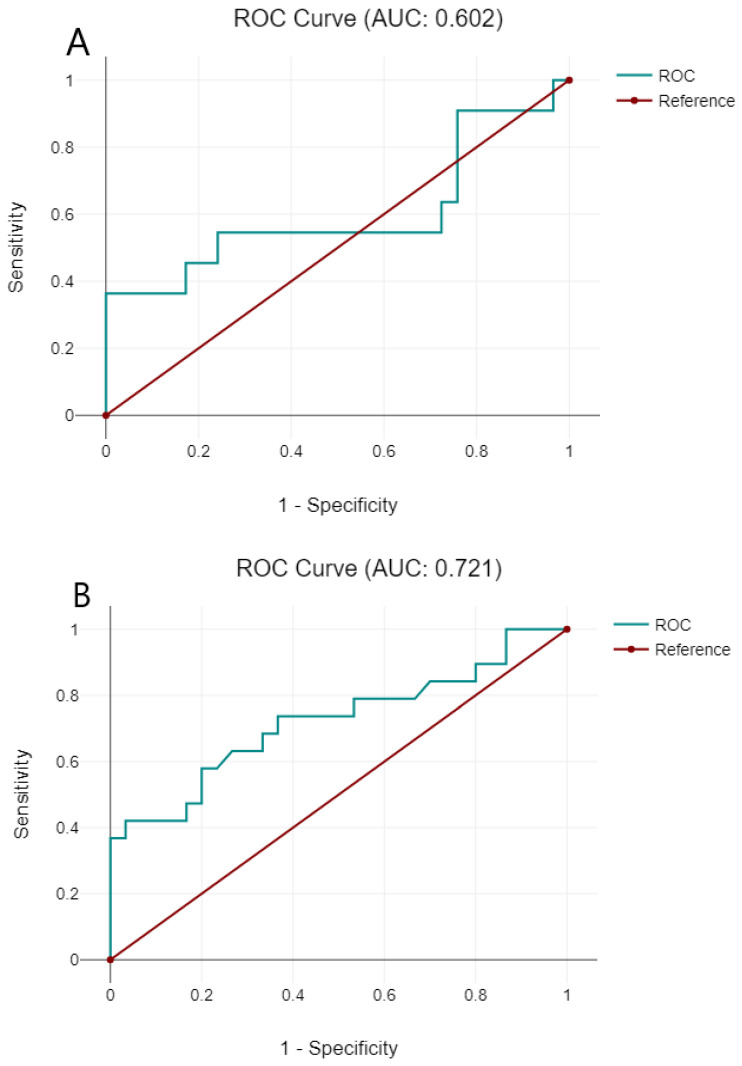
ROC curves of tumor volume reduction rate for differentiating good responders from bad responders in the chemotherapy group (**A**) and chemoradiotherapy group (**B**).

ROC curves were constructed to evaluate the value of TVRR for differentiating good responders from bad responders in the CT and CRT groups (Figure 4), which showed good sensitivity and specificity in predicting mrTRG.

### 3.4. Therapeutic Significance of TVRR, mrTRG, and pTRG in Anticipating Treatment Outcomes

A significant interaction in both CT and CRT groups showed that mrTRG, combined with tumor volume and VRR, is crucial in influencing the dependent variable, indicating that the synergy of mrTRG and volumetric measures is predictive of tumor response to treatment. This interaction, particularly in the CRT group, was markedly pronounced, indicating that combining tumor physical response metrics with regression grading is pivotal in assessing therapeutic efficacy.

In the CT group, the Pearson correlation coefficient analysis revealed a very high positive relationship between mrTRG and pTRG, with a correlation value (r) of 0.74, which indicates a strong association, where higher mrTRG scores are likely to correspond with higher pTRG scores within this sample. The statistical significance of this correlation was confirmed, with a *p*-value of less than 0.001. Similarly, in the CRT group, the Pearson correlation analysis demonstrated a very high positive correlation between mrTRG and pTRG, with an even stronger correlation coefficient (r) of 0.87. This also signifies a strong connection, suggesting that mrTRG is a reliable predictor of pTRG in this group. The association’s significance was statistically validated, with a *p*-value of less than 0.001. These results conclusively suggest that mrTRG is a potent predictive marker for pTRG in both treatment groups, which could potentially be used as a non-invasive method to estimate pathological response in patients undergoing treatment for rectal cancer.

ROC curves were constructed to evaluate the value of TVRR for differentiating good responders from bad responders in CT and CRT groups (Figure 4).

## 4. Discussion

There have been only a few similar studies in recent years, but this is the first study to examine the effect of two different treatment methods on the reduction in tumor volume. Our study involved 89 patients aged 37 to 83 years, predominantly male (67.4%), and focused on evaluating the clinical response to CT and CRT in rectal cancer. We used MRI scans and pathological assessments to stage tumors and observed significant tumor volume reduction and regression in both treatment groups. In the CT group, the pre- and post-therapy tumor volumes averaged 48.24 cm^3^ and 30.66 cm^3^, respectively, with a reduction rate of 36.52%. The CRT group showed similar initial volumes but a greater post-treatment reduction, averaging 20.54 cm^3^ and a reduction rate of 54.87%. The results demonstrate that both CT and CRT can effectively reduce tumor volumes. However, combination therapy (CRT) may lead to a more significant tumor volume reduction, especially in cases with a higher pathological tumor regression grade (pTRG 3/4). The data suggest that for certain patients, the synergistic effects of combining chemotherapy with radiotherapy could result in a more substantial tumor response, which can be critical for patient management and treatment efficacy. However, the lack of statistical significance in the post-therapy volumes within each group indicates the need for individualized treatment plans and consideration of other factors that might influence the therapeutic outcome.

In our analysis, we underscored the critical role of mrTRG when combined with tumor volumetrics, such as volume and VRR, in determining treatment response, especially in the CRT group. This interplay between tumor physical response and regression grading is key for evaluating therapeutic effectiveness. Based on this understanding, modern studies are increasingly focusing on developing new methods to improve the use of post-treatment MRI for predicting pathological responses to preoperative treatments in patients with locally advanced rectal cancer [14]. This trend highlights a growing interest in enhancing the predictive capabilities of MRI in this context [6].

While there were studies with a fair agreement in the correlation between mrTRG and pTRG [4,15,16], we found a very high positive correlation between mrTRG and pathological tumor regression grade (pTRG) in both treatment groups, establishing mrTRG as a reliable non-invasive predictor of pathological response in rectal cancer treatment.

TVRR has been identified as a potent predictor of the effectiveness of neoadjuvant chemotherapy for rectal cancer [17]. In our observations, TVRR stands out for its high sensitivity and specificity, making it adept at differentiating various levels of treatment responses. Notably, we have seen that a higher TVRR is often associated with a range of positive outcomes. This includes improved tumor differentiation, better tumor regression grades, and more significant T downstaging. This pattern suggests that TVRR could be a valuable tool in understanding and predicting treatment efficacy [18]. In addition, overall downstaging in rectal cancer, which encompasses a reduction in both the local extent and nodal involvement of the tumor, is associated with a greater TVRR. The correlation between TVRR and clinical outcomes has profound implications. Patients exhibiting a significant reduction in tumor volume typically have better prognoses. This information informs immediate treatment strategies and plays a crucial role in shaping post-operative care and surveillance approaches [19]. TVRR serves as an important tool for clinicians to customize follow-up care for rectal cancer patients. Depending on the degree of tumor volume reduction observed, it allows for adjustments in the frequency and intensity of post-treatment surveillance [20]. Additionally, TVRR provides valuable insights into tumor biology, informing future therapeutic strategies and research in rectal cancer treatment. Understanding the specific patterns and rates of tumor volume reduction enables surgeons and oncologists to predict patient outcomes more accurately and tailor treatment protocols to meet each patient’s individual needs.

Furthermore, tumor regression grade has been identified as a prognostic factor for disease-free survival, local failure, metastasis-free survival, and overall survival in patients receiving preoperative CRT for rectal cancer [20,21,22]. This discovery highlights the importance of initial tumor volume, volume reduction rate, and mrTRG as predictive tools in rectal cancer treatment. They are instrumental for surgeons in discussing long-term patient outcomes, guiding recovery, and setting expectations for future health. Recognizing how tumor characteristics interact with treatment responses is crucial in managing rectal cancer. However, the variability within treatment groups and the lack of significant changes in post-therapy volumes underscore the necessity for personalized treatment plans and the need to consider other factors that impact therapeutic results [23].

Our study has several limitations that need to be considered. First, the sample size of 89 patients, while substantial, is relatively modest for clinical research, potentially limiting the statistical power and generalizability of the findings, particularly given the skewed gender distribution favoring male patients. Second, the tumor boundaries on each image slice were manually traced to define an irregular region of interest (ROI). Finally, it is a single-center patient cohort, and the results may not directly apply to different patient populations or healthcare settings.

While current metrics demonstrate significant potential as predictive tools in treating rectal cancer, further research is urgently needed. This research is essential to refine these metrics, explore the biological mechanisms influencing tumor behavior, and establish standardized protocols for clinical use. Improving our understanding and applying these metrics will enhance their predictive value, leading to better patient outcomes through more targeted and effective rectal cancer treatments.

The application of AI in healthcare data is another promising avenue, with the potential to revolutionize early cancer diagnosis and address capacity concerns through automation. However, challenges such as ensuring model validation, addressing data privacy issues, and overcoming biases must be addressed. Despite these challenges, AI’s rapid growth and potential benefits in healthcare are undeniable [24,25]. AI’s capability in volume calculation could be a game changer, offering precise and efficient analysis, thereby enhancing the accuracy of treatment response evaluations and prognosis in various cancer types, including rectal cancer. This integration of AI could be the key to unlocking more effective and personalized cancer treatment strategies in the future.

Finally, understanding the metrics of tumor volume, tumor volume reduction rate, and magnetic resonance tumor regression grade is crucial for fostering a multidisciplinary approach to rectal cancer care. This knowledge allows for effective collaboration among surgeons, medical oncologists, and radiation oncologists because they can make more informed decisions based on a comprehensive understanding of the tumor’s characteristics and its response to various treatments. Such collaboration leads to the development of cohesive and personalized treatment plans tailored to individual patients’ specific needs and response patterns [26].

## 5. Conclusions

While both CT and CRT effectively reduced tumor volumes, the CRT group, especially those with higher pTRG 3/4 (good responders), showed a more pronounced response. It suggests combining chemotherapy with radiotherapy could yield a more substantial tumor response, which is essential for patient management and treatment efficacy. Rectal tumor volume, volume reduction rate, and mrTRG are not just abstract measures; they are concrete indicators that directly and practically impact surgical decision-making, planning, and prognosis, ultimately influencing the quality of care and life expectancy of patients with rectal cancer.

## Figures and Tables

**Table 1:** Characteristics of patients (*n* = 89).

Characteristics	Chemotherapy Group (CT) Number (%)/Average (SD)	Control Chemoradiotherapy Group (CRT) Number (%)/Average (SD)
Number of patients	40 (44.9%)	49 (55.1%)
Gender Male Female	27 (67.5%) 13 (32.5%)	33 (67.3%) 16 (32.7%)
Age (SD)	61.5 (SD 8.805)	66.45 (SD 8.949)
Differentiation Well/Moderate Poor/mucinous adenocarcinoma	34 (85%) 6 (15%)	43 (87.7%) 6 (12.3)
cT Stage cT2 cT3 cT4	0 (0%) 34 (85%) 6 (15%)	3 (6.1%) 40 (81.6%) 6 (12.3%)
cN Stage N- N+	0 40	1 48
CEA	10.19 (SD 15.54)	8.48 (SD 10.37)
CA-19.9	20.46 (SD 23.53)	18.46 (SD 16.53)

c = clinical, cT = clinical stage before treatment; cN = clinical nodal staging before treatment; CEA = carcinoembryonic antigen; CA19-9 = carbohydrate antigen 19-9; SD = standard deviation.

**Table 2:** Magnetic resonance and pathological tumor regression grade.

Magnetic Resonance Tumor Regression Grade (mrTRG)		Pathological Tumor Regression Grade (pTRG)	
mrTRG5 (no response)	No regression (intermediate signal intensity, same appearances as original tumor)	pTRG0 (no response)	No regression
mrTRG4 (slight response)	Slight regression (little areas of low signal intensity fibrosis or mucin but mostly tumor)	pTRG1 (minimal response)	Dominant tumor mass with obvious fibrosis and/or vasculopathy
mrTRG3 (moderate response)	Moderate regression (low signal intensity fibrosis predominates, but there are obvious areas of intermediate signal intensity)	pTRG2 (partial response)	Dominantly fibrotic changes with tumor cells or groups (easy to find)
mrTRG2 (near-complete response)	Good regression (predominant low signal intensity fibrosis with no obvious residual tumor signal)	pTRG3 (near-complete response)	Very few (difficult to find microscopically) tumor cells in fibrotic tissue with or without mucous substance
mrTRG1 (complete response)	Complete regression (absence of tumor signal and barely visible treatment-related scar)	pTRG4 (complete response)	No tumor cells, only fibrotic mass (total regression or response)

**Table 3:** Tumor stage and clinical, radiological, and pathological distribution in the chemotherapy and chemoradiotherapy groups.

	Chemotherapy Group (CT) *n* = 40 (% in Group)			Control Chemoradiotherapy Group (CRT) *n* = 49 (% in Group)	
MRI T stage		cT	yT	cT	yT
	T0	-	2 (5)	-	4 (8.2)
	T1	-	-	-	-
	T2	-	6 (15)	3 (6.1)	-
	T3a	-	10 (25)	19 (38.8)	8 (16.3)
	T3b	16 (40)	19 (47.5)	14 (28.5)	37 (75.5)
	T3c	13 (32.5)	2 (5)	7 (14.3)	-
	T3d	5 (12.5)	1 (2.5)	6 (12.2)	-
	T4a	6 (15.0)	-	-	-
MRI N stage		cN	yN	cN	yN
	N0	-	15 (37.5)	1 (2)	19 (38.8)
	N1	13 (32.5)	11 (27.5)	17 (34.7)	27 (55.1)
	N2	27 (67.5)	14 (35.0)	31 (63.3)	3 (6.1)
MRI EMVI		cEMVI	yEMVI	cEMVI	yEMVI
	EMVI-	17 (42.5)	28 (70)	27 (55.1)	34 (69.4)
	EMVI+	23 (57.5)	12 (30)	22 (44.9)	15 (30.6)
MRI MRF		cMRF	yMRF	cMRF	yMRF
	MRF-	20 (50)	28 (70)	20 (40.8)	38 (77.6)
	MRF+	20 (50)	12 (30)	29 (59.2)	11 (22.4)
	Before treatment		After treatment	Before treatment	After treatment
CEA	10.19 (SD 15.54)		3.34 (SD 2.49)	8.48 (SD 10.37)	4.1 (SD 6.11)
CA-19.9	20.46 (SD 23.53)		14.79 (SD 11.58)	18.46 (SD 16.53)	22.98 (SD 73.69)

c = clinical, y = after treatment; cT = clinical stage before treatment; yT = stage after treatment; N = nodal staging; EMVI = extramural venous invasion; MRF = mesorectal fascia; CEA = carcinoembryonic antigen; CA19-9 = carbohydrate antigen 19-9; SD = standard deviation.

**Table 4:** Pathological and Volumetry distribution in the chemotherapy and chemoradiotherapy groups.

		Chemotherapy Group (CT) *n* = 40 (% in Group)	Control Chemoradiotherapy Group (CRT) *n* = 49 (% in Group)
pT stage	pT0	4 (10)	9 (18.4)
	pT1	1 (2.5)	2 (4.1)
	pT2	8 (20)	9 (18.4)
	pT3	27 (67.5)	29 (59.2)
pN stage	pN0	21 (52.5)	33 (67.3)
	pN1	15 (37.5)	12 (24.5)
	pN2	4 (10)	4 (8.2)
LVI (pathological)	LVI-	24 (60)	34 (69.4)
	LVI+	16 (40)	15 (30.6)
CRM (after resection)	CRM-	35 (87.5)	46 (93.9)
	CRM+	5 (12.5)	3 (6.1)
V _Pre-Therapy_ (cm^3^)		48.24 (SD 17.67)	46.97 (SD 16.45)
V _Post-Therapy_ (cm^3^)		30.66 (SD 13.78)	20.54 (SD 10.41)
TVRR (%)		36.52 (SD 17.01)	54.87 (SD 19.57)
mrTRG	1	3 (7.5)	7 (14.2)
	2	7 (17.5)	17 (34.7)
	3	18 (45)	24 (48.8)
	4	12 (30)	1 (2.3)
pTRG	1	5 (12.5)	1 (2.3)
	2	24 (60)	29 (59.2)
	3	7 (17.5)	13 (26.5)
	4	4 (10)	6 (12)

p = pathological; pT = pathological stage; pN = pathological nodal staging; LVI = lymphovascular invasion (pathological); CRM = circumferential resection margin; V Pre-Therapy (cm^3^) = tumor volume before treatment; V Post-Therapy (cm^3^) = tumor volume after treatment; TVRR (%) = tumor volume reduction rate; SD = standard deviation; mrTRG = magnetic resonance tumor regression grade; pTRG = pathological tumor regression grade.

**Table 5:** Tumor Volumetry Results according to mrTRG.

		Chemotherapy Group (CT) *n* = 40 (% in Group)		Control Chemoradiotherapy Group (CRT) *n* = 49 (% in Group)	
	mrTRG	good responders 1/2	bad responders 3/4/5	good responders 1/2	bad responders 3/4/5
Volumetry					
V _Pre-Therapy_ (cm^3^)		47.96 (SD 18.54)	48.98 (SD 15.95)	45.13 (SD 17.26)	48.89 (SD 15.7)
*p* Value		0.436		0.429	
V _Post-Therapy_ (cm^3^)		26.75 (SD 16.59)	32.14 (SD 12.56)	17.09 (SD 11.21)	23.89 (SD 8.54)
*p* Value		0.179		0.138	
TVRR (%)		48.05 (SD 23.65)	32.14 (SD 11.45)	64.88 (SD 19.89)	45.26 (SD 13.82)
*p* Value		<0.001		<0.001	

1/2 good responders; 3/4/5 bad responders; Pre-Therapy (cm^3^) = tumor volume before treatment; V Post-Therapy (cm^3^) = tumor volume after treatment; TVRR (%) = tumor volume reduction rate; SD = standard deviation; *p* value <0.05 for significant results.

**Table 6:** Tumor Volumetry Results according to pTRG.

		Chemotherapy Group (CT) *n* = 40 (% in Group)		Control Chemoradiotherapy Group (CRT) *n* = 49 (% in Group)	
	pTRG	good responders 3/4	bad responders 0/1/2	good responders 3/4	bad responders 0/1/2
Volumetry					
V _Pre-Therapy_ (cm^3^)		49.69 (SD 18.47)	47.68 (SD 17.66)	46.23 (SD 15.63)	47.45 (SD 17.2)
*p* Value		0.351		0.272	
V _Post-Therapy_ (cm^3^)		27.67 (SD 18.28)	31.79 (SD 11.85)	15.99 (SD 11.58)	23.43 (SD 8.59)
*p* Value		0.187		0.117	
TVRR (%)		46.18 (SD 25.36)	32.85 (SD 11.08)	65.28 (SD 21.18)	48.28 (SD 15.48)
*p* Value		<0.001		<0.001	

3/4 good responders; 0/1/2 bad responders; Pre-Therapy (cm^3^) = tumor volume before treatment; V Post-Therapy (cm^3^) = tumor volume after treatment; TVRR (%) = tumor volume reduction rate; SD = standard deviation; *p* value <0.05 for significant results.

## Data Availability

The data and materials used in this study are available upon reasonable request from the corresponding author. Restrictions may apply to the availability of certain data sets due to privacy or ethical restrictions.
